# Supplementary material for: HIVprotI: an integrated web based platform for prediction and design of HIV proteins inhibitors
Source: J Cheminform. 2018 Mar 9;10:12. doi: 10.1186/s13321-018-0266-y (PMC5845081; doi:10.1186/s13321-018-0266-y)
Supplement: Supplementary file 2 — Additional file 2. Source code of HIVProtI web server. [file 13321_2018_266_MOESM2_ESM.zip › HIVprotI_Source-code/hivprotI/weka-3-6-6/doc/weka/classifiers/functions/package-summary.html]

weka.classifiers.functions


---


|  |  |  |
| --- | --- | --- |
| **Overview** \| **Package** \| Class \| **Tree** \| **Deprecated** \| **Index** \| **Help** \| **Weka's home** | |  |
| **PREV PACKAGE**   **NEXT PACKAGE** | **FRAMES**    **NO FRAMES**     **All Classes** |  |


---

## Package weka.classifiers.functions

| **Class Summary** | |
| --- | --- |
| **GaussianProcesses** | Implements Gaussian Processes for regression without hyperparameter-tuning. |
| **IsotonicRegression** | Learns an isotonic regression model. |
| **LeastMedSq** | Implements a least median sqaured linear regression utilising the existing weka LinearRegression class to form predictions. |
| **LibLINEAR** | A wrapper class for the liblinear tools (the liblinear classes, typically the jar file, need to be in the classpath to use this classifier).  Rong-En Fan, Kai-Wei Chang, Cho-Jui Hsieh, Xiang-Rui Wang, Chih-Jen Lin (2008). |
| **LibSVM** | A wrapper class for the libsvm tools (the libsvm classes, typically the jar file, need to be in the classpath to use this classifier).  LibSVM runs faster than SMO since it uses LibSVM to build the SVM classifier.  LibSVM allows users to experiment with One-class SVM, Regressing SVM, and nu-SVM supported by LibSVM tool. |
| **LinearRegression** | Class for using linear regression for prediction. |
| **Logistic** | Class for building and using a multinomial logistic regression model with a ridge estimator.    There are some modifications, however, compared to the paper of leCessie and van Houwelingen(1992):     If there are k classes for n instances with m attributes, the parameter matrix B to be calculated will be an m\*(k-1) matrix.    The probability for class j with the exception of the last class is    Pj(Xi) = exp(XiBj)/((sum[j=1..(k-1)]exp(Xi\*Bj))+1)     The last class has probability    1-(sum[j=1..(k-1)]Pj(Xi))   = 1/((sum[j=1..(k-1)]exp(Xi\*Bj))+1)    The (negative) multinomial log-likelihood is thus:     L = -sum[i=1..n]{  sum[j=1..(k-1)](Yij \* ln(Pj(Xi)))  +(1 - (sum[j=1..(k-1)]Yij))   \* ln(1 - sum[j=1..(k-1)]Pj(Xi))  } + ridge \* (B^2)    In order to find the matrix B for which L is minimised, a Quasi-Newton Method is used to search for the optimized values of the m\*(k-1) variables. |
| **MultilayerPerceptron** | A Classifier that uses backpropagation to classify instances.  This network can be built by hand, created by an algorithm or both. |
| **PaceRegression** | Class for building pace regression linear models and using them for prediction. |
| **PLSClassifier** | A wrapper classifier for the PLSFilter, utilizing the PLSFilter's ability to perform predictions. |
| **RBFNetwork** | Class that implements a normalized Gaussian radial basisbasis function network.  It uses the k-means clustering algorithm to provide the basis functions and learns either a logistic regression (discrete class problems) or linear regression (numeric class problems) on top of that. |
| **SimpleLinearRegression** | Learns a simple linear regression model. |
| **SimpleLogistic** | Classifier for building linear logistic regression models. |
| **SMO** | Implements John Platt's sequential minimal optimization algorithm for training a support vector classifier.    This implementation globally replaces all missing values and transforms nominal attributes into binary ones. |
| **SMOreg** | SMOreg implements the support vector machine for regression. |
| **SPegasos** | Implements the stochastic variant of the Pegasos (Primal Estimated sub-GrAdient SOlver for SVM) method of Shalev-Shwartz et al. |
| **VotedPerceptron** | Implementation of the voted perceptron algorithm by Freund and Schapire. |
| **Winnow** | Implements Winnow and Balanced Winnow algorithms by Littlestone.    For more information, see    N. |

---


|  |  |  |
| --- | --- | --- |
| **Overview** \| **Package** \| Class \| **Tree** \| **Deprecated** \| **Index** \| **Help** \| **Weka's home** | |  |
| **PREV PACKAGE**   **NEXT PACKAGE** | **FRAMES**    **NO FRAMES**     **All Classes** |  |


---
